# Acute macular neuroretinopathy after Covid 19 in an eye with a congenital retinal macrovessel – a case report

**DOI:** 10.1016/j.ajoc.2025.102349

**Published:** 2025-05-06

**Authors:** A. Vienne-Jumeau, E. Souied, F. Amoroso

**Affiliations:** a Centre Hospitalier National d'ophtalmologie des Quinze-Vingts, Paris, France; b Borelli Centre, UMR 9010, CNRS-SSA-ENS Paris Saclay-Université Paris Cité, 91190, Paris, France; c Department of Ophthalmology, University of Paris Est-Creteil, Créteil, France

**Keywords:** Acute macular neuroretinopathy, Covid 19, Macrovessel, Retinal capillary occlusion

## Abstract

**Purpose:**

To present a case of an acute macular neuroretinopathy (AMN) occurring after Covid 19 infection in an eye with a retinal macrovessel.

**Observations:**

We report a case of a 48-year-old woman who presented to our center with a 10-day scotoma in her right eye, in the context of a recently diagnosed Covid 19 infection. The funduscopy demonstrated a petaloid brown lesions temporal to the fovea in the right eye. A retinal macrovessel, passing adjacent to the lesions was also present. The left eye was normal. Multimodal imaging confirmed the diagnostic of AMN and optical coherence tomography angiography showed defects in the deep capillary plexus colocalizing with the lesions.

**Conclusions and importance:**

Ischemic events have been known to complicate macrovessel, and we reckon that the occurrence of AMN in this patient could have been the resultant of the conjunction of Covid 19 infection and abnormal retinal vasculature.

## Teaser

**Claims of Priority statement:** After conducting a literature review on September 20, 2024, using PubMed, Google Scholar, and Medline with the keywords "macrovessel" and ("macular neuroretinopathy" or "AMN"), we found no prior reports of the concomitant occurrence of a congenital macrovessel and macular neuroretinopathy.

## 1 Introduction

Acute macular neuroretinopathy (AMN) is a rare retinal disease of unknown etiology, first described by Bos and Deutman in 1975,^1^ which primarily affects young and middle-aged women.^2^ Flu-like prodromes are commonly reported, and the disease typically manifests with paracentral scotomas. Bilateral involvement occurs in approximately half of the cases.^2^ Recent studies suggest that ischemia of the deep retinal capillary plexus may underlie the characteristic wedge-shaped lesions seen in AMN.^3^ While the exact pathophysiology of this ischemia remains debated, several risk factors have been identified, including flu-like illnesses—particularly COVID-19 infection^4^^,^^5^— prior blunt trauma,^6^ the use of oral contraceptives or vasoconstrictive agents (such as epinephrine or ephedrine), systemic shock,^7^ and retinal vascular tortuosity.^8^ These factors may compromise retinal capillary perfusion. Additionally, there have been reports of concomitant paracentral acute middle maculopathy (PAMM), suggesting that both conditions may share common etiologies or risk factors.^2^^,^^9^

Congenital retinal macrovessels (CRM) are dilated retinal vessel supplying or draining the macula, both inferior and superior to the horizontal raphe, first described by Mauthner in 1869.^10^ While most CRMs are venous, arterial CRMs have also been reported.^11^ Although often asymptomatic and discovered incidentally, complications can arise, such as macular edema, ischemic occlusions, and intravitreal or macular hemorrhages.^2^^,^^12^ Angiographic studies have shown that some CRMs are associated with perifoveal areas of capillary non-perfusion.^13^

Herein, we describe a case of AMN in an eye with CRM and discuss the potential role of the CRM in the pathophysiology of the AMN.

## 2 Case

**Fig. 1:**
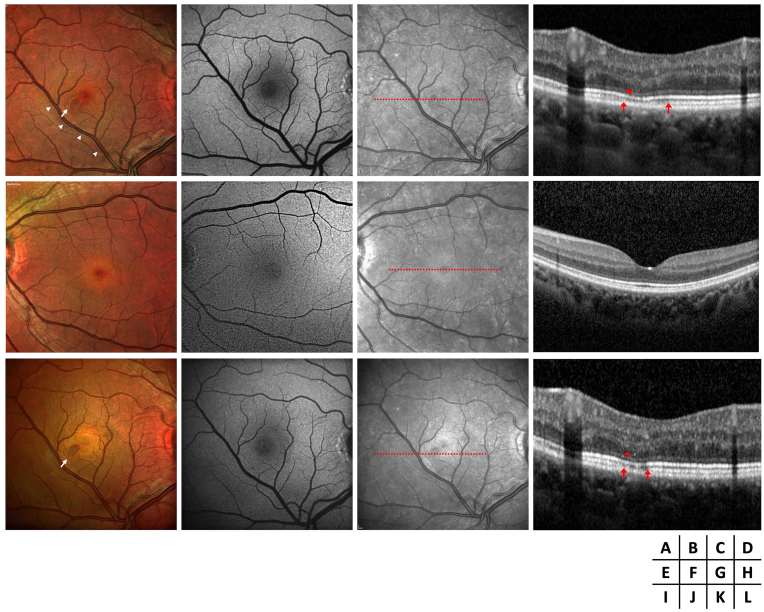
Multimodal imaging of the right eye (A–D) and left eye (E–H) at diagnosis, and of the right eye after one year (I–L), includes retinophotography, fundus blue autofluorescence (BAF), near-infrared (NIR), and optical coherence tomography (OCT). Retinophotography of the right eye reveals a reddish-brown, petaloid-shaped lesion temporal to the fovea (white arrow), along with an inferotemporal dilated retinal vein crossing the horizontal raphe, suggestive of a congenital retinal macrovessel (CRM) (white arrowhead) (A). The petaloid lesion appears normal on BAF (B) but hyporeflective on NIR (C). The OCT B-scan through the lesion shows hyperreflectivity in the outer plexiform and outer nuclear layers, with crumbling and attenuation of the ellipsoid zone (red arrowhead) and interruption of the interdigitation zone temporal to the fovea (delimited by red arrows) (D). Multimodal imaging of the left eye is normal (E–H). On follow-up, retinophotography, BAF, and NIR (I–K) do not show significant changes. The extent of the ellipsoid and interdigitation zone disruption is reduced on the OCT B-scan (L). (For interpretation of the references to colour in this figure legend, the reader is referred to the Web version of this article.)

A 48-year-old female presented to the ophthalmology department with a 10-day history of a grey oval spot in her visual field. Her medical history was unremarkable, except for a COVID-19 infection one month before symptom onset, confirmed by quantitative polymerase chain reaction on a nasal swab sample. The patient had followed the standard vaccination protocol for COVID-19 in France at that time, receiving two doses of the mRNA vaccine. She received the two-dose series, respectively, four and two months before symptom onset. The patient had been using oral estroprogestative contraception for more than ten years. Her visual acuity was 20/20 in both eyes, with normal intraocular pressure and an unremarkable anterior segment examination. Fundus examination of the right eye revealed a reddish-brown, petaloid-shaped lesion temporal to the fovea and an inferotemporal dilated retinal vein crossing the horizontal raphe, suggestive of a congenital retinal macrovessel (CRM) (Fig. 1A). Fundus blue autofluorescence (BAF) was normal (Fig. 1B), while near-infrared (NIR) imaging showed the lesion as hyporeflective with otherwise normal findings (Fig. 1C). Macular optical coherence tomography (OCT) B-scan demonstrated hyperreflectivity in the outer plexiform and outer nuclear layers, with crumbling and attenuation of the ellipsoid zone and interruption of the interdigitation zone temporal to the fovea (Fig. 1D). Multimodal imaging of the left eye was normal (Fig. 1E–H).

**Fig. 2:**
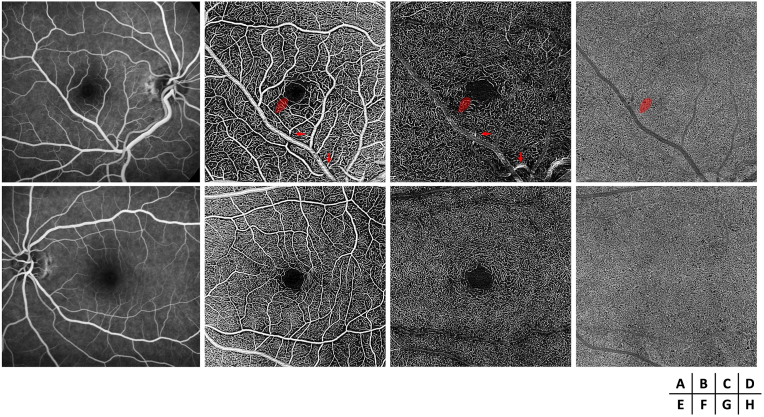
Fluorescein angiography (FA) and optical coherence tomography angiography (OCTA) of both eyes at diagnosis. The red mask indicates the location of the petaloid lesion. FA shows the venous macrovessel in the right eye, along with tortuosity and irregularity in the caliber of the second-order artery supplying the temporofoveal region, with localized narrowing (red arrows) at its origin and increased tortuosity upstream of its crossing with the CRM (A). OCTA reveals a localized decrease in perfusion in the deep capillary plexus in the temporofoveal region of the right eye (C), without a corresponding decrease in the superficial capillary plexus (B) or the choriocapillaris (D). FA and OCTA of the left eye is normal (E–H). (For interpretation of the references to colour in this figure legend, the reader is referred to the Web version of this article.)

**Fig. 3:**
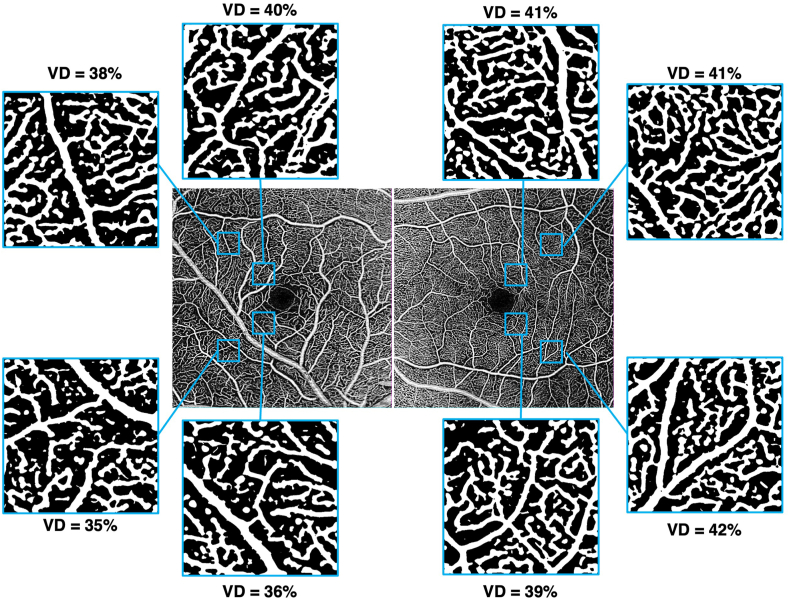
Vascular density (VD) in the superficial capillary plexus at four temporal parafoveal locations (superotemporal at 1000 μm and 2500 μm, and inferotemporal at 1000 μm and 2500 μm from the fovea) in the right eye (left panels) and left eye (right panels). The difference between the two eyes is not statistically significant.

**Fig. 4:**
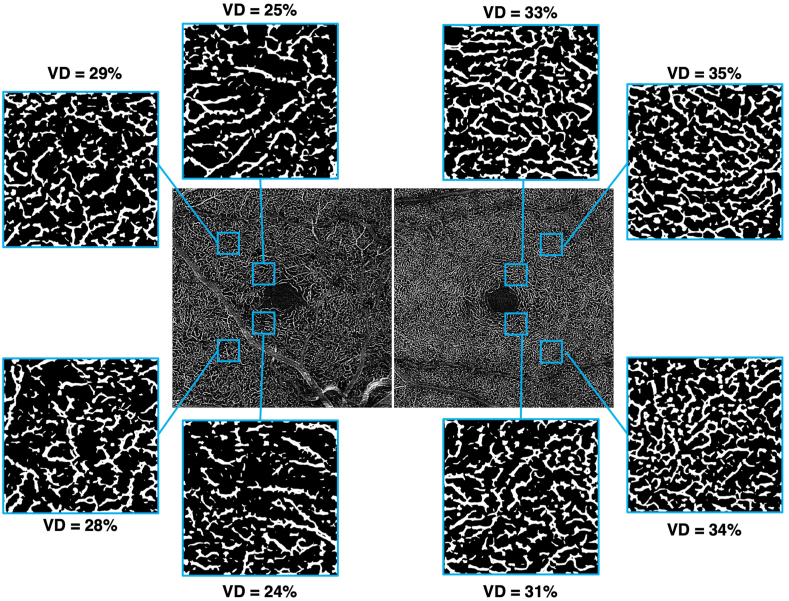
Vascular density (VD) in the deep capillary plexus at four temporal parafoveal locations (superotemporal at 1000 μm and 2500 μm, and inferotemporal at 1000 μm and 2500 μm from the fovea)in the right eye (left panels) and left eye (right panels). There is a decreased VD in the right eye compared to the left eye.

Fluorescein angiography during the venous phase confirmed the venous origin of the CRM, with no leakage (Fig. 2A). The second-order artery supplying the temporofoveal region, where the lesion was located, appeared irregular in caliber and tortuous, with localized narrowing at its origin and increased tortuosity upstream of its crossing with the CRM. OCT angiography (OCTA) of the right eye revealed a preserved superficial capillary plexus (SCP) temporal to the fovea (Fig. 2B) but an apparent reduction in vascular density (VD) was noted at the deep capillary plexus (DCP) level (Fig. 2C). The choriocapillaris was normal (Fig. 2D), as were all retinal layers in the left eye (Fig. 2E–H). Quantitative measurements of vascular density (VD) in four regions of interest within the temporal SCP and DCP are presented in Fig. 3, Fig. 4, respectively. At the SCP level, the mean VD in these regions was 9 % lower in the right eye compared to the left (Fig. 3: 37 % in the right eye vs. 41 % in the left eye, paired *t*-test, *p* = 0.07). In contrast, a more pronounced reduction was observed at the DCP level (Fig. 4), where the mean VD was 20 % lower in the right eye than in the contralateral eye (26 % vs. 33 %, paired *t*-test, *p* = 0.0008).

The diagnosis of acute macular neuroretinopathy (AMN) in an eye with previously undiagnosed CRM was made, and observation was recommended. One year later, the relative scotoma remained stable with no new symptoms. Follow-up funduscopy, BAF, and NIR imaging showed no progression of the lesion (Fig. 1I–L). OCT B-scan revealed partial recovery of the ellipsoid and interdigitation zones at the lesion's border (Fig. 1L).

## 3 Discussion

Retinal ischemic episodes are recognized as potential complications associated with congenital retinal macrovessels (CRM).^12^ Here, we report a rare case of acute macular neuroretinopathy (AMN) occurring in conjunction with CRM in the context of a COVID-19 infection.

In a series by Pichi et al.^11^ analysing 49 patients with CRM, two patients with unilateral AMN and Dengue fever were incidentally diagnosed with CRM in the same eye. AMN is a known manifestation of dengue maculopathy,^14^ and the role of CRM was only putative in these cases.

Both CRM and AMN share a common feature: capillary anomalies. Indeed, AMN is primarily characterized by localized reduced blood flow in the deep capillary plexus (DCP).^15^ Similarly, there are several reports of capillary hypoperfusion in association with CRM in several eyes within their cohort.^11^^,^^13^ Here, we also observed a decrease in DCP density in the eye affected by CRM, consistent with previous findings. Reduced perfusion was noted in both the superior and inferior regions temporal to the macula, with a more pronounced decrease in the temporoinferior region near the fovea, where the lesion occurred.

Although the precise mechanism linking CRM to such hypoperfusion has not been fully elucidated, one plausible hypothesis is that the CRM compresses adjacent structures, thereby reducing the flow of blood to the capillaries. In our case, the inferotemporal artery exhibits a tortuous path with irregular caliber with narrowing upstream to its crossing of the CRM, suggesting possible mechanical stress and compromised blood flow. Besides, the nasal part of the macula receives blood supply from both the superotemporal and inferotemporal arteries, while the temporal and inferior regions are primarily perfused by the inferotemporal artery. Notably, the AMN lesion in this patient is located temporal to the macula, which is atypical compared to the more commonly described locations.^1^ In fact, a review by Bhavsar et al. found that 14 out of 17 (82 %) lesions preferentially situated in the nasal or temporal regions are more commonly located in the nasal quadrant (case one to five of the case series by Sarraf et al. were paracentral acute middle maculopathy and were therefore not included in this statistics).^2^

We hypothesize that the CRM contributes to abnormal microvasculature at the level of the DCP, making it vulnerable to ischemia during vasogenic or pro-ischemic events, such as a COVID-19 infection. Further studies examining the response of these capillaries to various stressors could provide crucial insights into the underlying mechanisms of this association.

Importantly, the patient had been using oral oestroprogestative contraception for more than a decade. This type of contraception has been linked to an increased risk of AMN, likely due to its prothrombotic effects, which can contribute to microvascular ischemic events. Given this potential association, long-term use may have been a contributing factor in this case.

CRM can sometimes be associated with underlying cerebral venous malformations or other neurovascular anomalies. The study by Pichi et al.^11^ found a strong correlation between CRM and cerebral vascular malformations, particularly venous malformations, reporting that 24 % of patients with CRM were diagnosed with such anomalies. Based on these findings, the authors recommend performing a brain MRI with contrast to rule out central nervous system venous malformations in patients with CRM. Following this recommendation, the MRI was discussed with the patient and proposed as an examination. However, she declined it due to the absence of symptoms.

## CRediT authorship contribution statement

**A. Vienne-Jumeau:** Writing – review & editing, Writing – original draft, Visualization, Validation, Investigation, Data curation, Conceptualization. **E. Souied:** Writing – review & editing, Supervision. **F. Amoroso:** Writing – review & editing, Supervision.

## Patient consent

Consent to publish this case report has been obtained from the patient.

## Disclosure of interest

The authors have no financial disclosures.

## Declaration of competing interest

The authors declare that they have no known competing financial interests or personal relationships that could have appeared to influence the work reported in this paper.
